# Importance of early detection and treatment of occupational hypersensitivity pneumonitis

**DOI:** 10.1093/joccuh/uiaf026

**Published:** 2025-05-20

**Authors:** Shinya Ohkouchi, Yasuo Morimoto, Narufumi Suganuma, Hajime Kurosawa, Kenichi Azuma, Hisamitsu Omori, Taro Tamura, Kunio Dobashi, Kengo Nakamoto, Makiko Nakano, Yuji Natori, Naomi Hisanaga, Kiyoshi Mizushima, Kazuhiro Yatera, Yasunari Miyazaki, on behalf of Japan Society for Occupational Health, Occupational Lung Disease Study Group

**Affiliations:** Occupational Health, Graduate School of Medicine, Tohoku University, Sendai 980-8575, Japan; Department of Occupational Pneumology, Institute of Industrial Ecological Sciences, University of Occupational and Environmental Health, Japan, Kitakyusyu 807-8555, Japan; Department of Environmental Medicine, Kochi Medical School, Kochi, 783-8505, Japan; Occupational Health, Graduate School of Medicine, Tohoku University, Sendai 980-8575, Japan; Department of Preventive Medicine and Behavioral Sciences, Kindai University, Osaka-Sayama 589-0014, Japan; Graduate School of Health Sciences, Kumamoto University, Kumamoto 862-0976, Japan; Department of Environmental Medicine, Kochi Medical School, Kochi, 783-8505, Japan; Jobu Hospital for Respiratory Disease, Maebashi 371-0048, Japan; Nakamoto Industrial Health Consultant and Occupational Physician Office, Nagakute 480-1138, Japan; Chemical Safety Research Group, National Institute of Occupational Safety and Health, Kawasaki 214-8585, Japan; Himawari Clinic, Tokyo 136-0071, Japan; CKD Corporation, Komaki 485-8221, Japan; Mizushima Clinic, Higashi- Osaka 5770054, Japan; Department of Respiratory Medicine, University of Occupational and Environmental Health, Japan, Kitakyusyu 807-8555, Japan; Department of Respiratory Medicine, Graduate School of Medicine, Institute of Science Tokyo, Tokyo 1138519, Japan

**Keywords:** hypersensitivity pneumonitis, occupational, prevention

## Abstract

Recently, the incidence of pneumoconiosis has decreased due to strict dust control measures and environmental improvements in the workplace. The significance of other occupational diseases has relatively increased. Occupational hypersensitivity pneumonitis (OHP) is mainly caused by allergic reactions to antigens in the workplace. Therefore, the presence of subtle amounts of harmful substances in the environment can increase the risk of developing OHP. Not only organic substances but also inorganic substances can cause OHP. OHP is caused by a specific antibody reaction (type III allergy) or sensitized lymphocytes (type IV allergy) to a specific antigen. Based on the clinical course, OHP is classified into acute and chronic hypersensitivity pneumonitis (HP). Acute HP forms granulomas and is classified as a granulomatous lung disease (nonfibrotic HP), whereas chronic HP rarely forms granulomas and progresses to fibrosis (fibrotic HP). Differentiation between chronic HP and idiopathic or collagen vascular disease-related interstitial pneumonia is challenging. Additionally, the genetic background of each patient influences the onset, leading to diverse onset patterns. Antigens and modes of onset are diverse in the workplace. Therefore, diagnosis is difficult, and many patients may be misdiagnosed. Chronic HP with advanced fibrosis due to delayed antigen identification has a poor prognosis. This study aimed to present an overview of the causative antigens, diagnosis, prevention, and treatment of OHP to provide appropriate and timely medical attention.

## Teaser

Key points:

- The incidence of death from interstitial pneumonia is increasing. In Japan, interstitial pneumonia was the 11th leading cause of death in 2022, accounting for 22 905 (1.5%) of 1 569 050 deaths.
- Half of the deaths from interstitial pneumonia are attributed to idiopathic pulmonary fibrosis (IPF). However, some reports have shown that more than half of the cases previously considered IPF are now considered chronic hypersensitivity pneumonitis (HP) (fibrotic HP).
- Many HP cases are of occupational origin. Acute HP (nonfibrotic HP) is relatively easy to diagnose. Conversely, chronic HP progresses insidiously, and patients with advanced fibrosis have a poor prognosis.
- With a significant decline in the incidence of pneumoconiosis in industry due to workplace environmental improvements, the relative significance of occupational HP (OHP) has increased. OHP can present in both acute and chronic forms.
- HP is an allergic disease that can develop and progress due to the inhalation of small amounts of antigens. Therefore, occupational health specialists should have a comprehensive understanding of OHP.
- This study provides an overview of the concept, prevention, and treatment of OHP.

## 1. Disease concept of occupational hypersensitivity pneumonitis (OHP)

Hypersensitivity pneumonitis (HP) is an allergic or immune disease caused by repeated inhalation of antigens leading to inflammation.^1^^-^^4^ It is mainly characterized by lymphocyte infiltration of the alveolar septa and bronchioles. According to the ATS/JRS/ALAT (American Thoracic Society, Japanese Respiratory Society, Latin American Thoracic Association) guidelines, HP is “an inflammatory and/or fibrotic disease affecting the lung parenchyma and small airways.”^2^ It typically results from an immune-mediated reaction to overt or occult inhaled antigens in susceptible individuals.^3^ Generally, acute HP often ends with inflammation alone, whereas chronic HP becomes complicated by fibrosis. Inhaled antigens in the environment have been detected in occupational, bird-related, and home-related HP cases. If symptoms occur in the workplace, it can be diagnosed as OHP. If the specific antigen that causes the symptoms is not identified in the workplace, it is difficult to determine whether the cause is at work or in the environment, such as at home. In Japan, the major types of HP include summer-type, home-related, bird-related, farmer’s lung, painter’s lung, humidifier lung, and mushroom grower’s lung. Farmer’s lung, malt worker’s lung, and maple bark disease have long been known as acute lung diseases associated with certain occupations.^5^^,^^6^

In 1959, Pepys et al identified anti-*Aspergillus* antibodies in the serum of patients with malt worker’s lung, and precipitated antibodies against thermophilic actinomycetes in the serum of patients with farmer’s lung; malt worker’s lung was recognized as an allergic disease. The incidence of OHP is estimated to be 0.4–2.7 per million workers in the United Kingdom. A previous study using a UK database from 1996 to 2015 showed that machine operator’s lung (35%) was the most common, followed by farmer’s lung (17%) and bird-related HP (11%). This study showed that HP increases in workplace environments with high mist and humidity levels.^7^

Many Japanese studies have shown that the number of patients with farmer’s lung is decreasing (Table 1).^5^^,^^6^^,^^8^^,^^9^ In the case of acute HP, farmer’s lung accounted for 8.1% of all cases in 1980-1989 and 4.4% in 1990-1999. In the case of chronic HP, farmer’s lung accounted for 11.1% of all cases in 1989-1998. From 2001 to 2009, this percentage decreased to 1.8%. Yoshida et al conducted a Japanese national survey of OHP from 1980 to 1989 and reported that 69% of the patients with OHP were farmers, and most of them had farmer’s lung. Occasionally, greenhouse or mushroom grower’s lung was observed in farmers. The following most common types of OHP were observed in workers exposed to chemicals. In this category, painter’s lung caused by diisocyanates was common.^10^ The incidence of these diseases seems to decrease due to improvements in work environments and health management in the workplace and community.

**Table 1:** Epidemiological data for acute and chronic hypersensitivity pneumonitis.

**Acute hypersensitivity pneumonitis**					**Chronic hypersensitivity pneumonitis**						**Hypersensitivity pneumonia DPC data**		
	**1980-1989** ^**a**^		**1990-1999** ^**b**^			**1989-1998** ^**c**^			**2001-2009** ^**d**^			**2011-2017** ^**e**^	
**Disease**	**No. of cases**	**%**	**No. of cases**	**%**	**Disease**	**No. of cases**	**%**	**Disease**	**No. of cases**	**%**	**Disease**	**No. of cases**	**%**
Summer type	621	74.4	624	69.8	Summer type	10	27.8	Bird-related	134	60.4	Summer type	490	13.5
Famer’s lung	68	8.1	53	5.9	Bird-related	7	19.4	Summer type	33	14.9	Bird-related	199	5.5
Humidifier lung	36	4.3	39	4.4	Isocyanate	5	13.9	Home-related	25	11.3	Humidifier lung	106	2.9
Bird-related	34	4.1	36	4	Home-related	5	13.9	Farmer’s lung	4	1.8	Famer’s lung	48	1.3
Others	19	2.3	68	7.6	Famer’s lung	4	11.1	Isocyanate	3	1.4	Others	2761	76
Unknown	57	6.8	74	8.3	Others	5	13.9	Others	23	10.4	Total	3634	100
Total	835	100	894	100	Total	36	100	Total	222	100			

Abbreviation: DPC, Diagnostic Procedure Combination database (a large nationwide database in Japan).

a *American Review of Respiratory Disease* 1991; 144: 765-9.

b *Nihon-kyoubu-rinnsyou* 2003; 62-2: 97-106 [in Japanese].

c *Journal of Allergy and Clinical Immunology* 1999; 103: 315-20.

d *Respiratory Investigation* 2013; 51: 191-9.

e *Respiratory Investigation* 2023; 61:172.

The Japan Agency for Medical Research and Development is conducting a new epidemiological survey of HP in Japan from 2022, which is expected to reveal the latest information on the prevalence, morbidity, frequency of fibrotic complications, and causative antigens of OHP in Japan.

## 2. Pathophysiology

### 2.1. Environmental factors and causative antigens

Specific occupations are prone to the development of OHP. The causative antigens are diverse, including bacteria, fungi, animal-derived proteins (heterologous proteins), and chemicals. Table 2 shows the causative antigens for each occupation, and Table 3 shows the classification by source. Acute OHP is more common because workers are often exposed to a large amount of antigen in the workplace, whereas chronic OHP with acute symptoms has been reported in farmers’ lung and painter’s lung cases. In Western countries, thermophilic actinomycetes are the main antigens responsible for mushroom grower’s lung. In Japan, the spores of shiitake, shimeji, nameko, and eringi mushrooms are the causes. Recently, the number of machine operator’s lung cases has increased; this is caused by *Acinetobacter*, *Ochrobactrum*, and *Mycobacterium immunogenum* in metal-cleaning solutions (synthetic water-soluble machine washing) used in auto parts factories. Regarding the classification of OHP by cause, bird-related HP is the most common type, followed by farmer’s lung, summer-type HP, and home-related HP.^7^ In Japan, fungi, mainly *Trichosporon asahii* (summer-type HP), are the most common cause of acute HP (74%). Conversely, in chronic HP, fungi account for approximately 25% of the cases, and bird-related antigens account for approximately 60% of the cases.^5^^,^^6^ In other words, HP caused by fungi is often acute, whereas HP caused by bird-related antigens is often chronic. It is assumed that immune responses differ based on the nature of the antigen. However, the detailed mechanism remains unknown.^3^

**Table 2:** Antigens causing occupational hypersensitivity pneumonitis.

**Type of antigen**	**Disease and occupation**	**Onset environment**	**Antigen**
**Bacterial**	Farmer’s lung	Dairy farming work	*Saccharopolyspora rectivirgula, Thermoactinomyces vulgaris, Absidia corymbifera, Eurotium amstelodami, Wallemia sebi*
		
		
		
		Tractor driving	*Rhizopus* genus
	Machine operator’s lung	Synthetic water-soluble machine washing	*Acinetobacter, Ochrobactrum, Mycobacterium immunogenum*
	
	Spa employees	Mist	*Mycobacterium avium* complex
	Hot tub lung	Hot tub, shower, mist	*M. avium* complex
	Humidifier lung, air conditioner lung	Humid working environment	*Klebsiella oxytoca*
	Food waste cleaning	Food waste cleaning	*T. vulgaris*
	Sugarcane lung	Sugarcane fields	*T. vulgaris*
**Fungal**	Mushroom grower’s lung	Shiitake cultivation	Shiitake mushroom spores, enoki mushroom spores
		Enoki cultivation	
	Humidifier lung, air conditioner lung	Humid working environment	*Alternaria alternata, Aspergillus flavus, Phoma herbarum, Rhodotorula*
	
	Cork lung	Cork production	*Penicillium glabrum, Aspergillus fumigatus, Chrysonilia sitophila*
		
		
	Maple bark disease	Maple crop production	*Cryptostroma corticale*
	Wine production	Mold in grapes	*Botrytis cinerea*
	Cheese manufacturing	Mold in cheese	*Penicillium roqueforti*
	Miso and soy sauce manufacturing		*Aspergillus oryzae*
Greenhouse grower’s lung	Orchid, cucumber cultivation (greenhouse)	*A. fumigatus*	*A. fumigatus*
	Traditional craftsmen	*Zizania latifolia* ink	*Ustilago esculenta*
	Summer-type HP	Mold growing on rotting wood	*Trichosporon asahii, Trichosporon mucoides*
**Animal**	Bird-related HP	Bird breeding	Avian droppings, serum components
		Field work	Manure fertilizer
		Making taxidermy	Feathers
		Duvet manufacturing	Feathers
	Researcher	Animal experiments	Rat serum
**Small molecule chemicals**	Painter’s lung	Automobile paint, piano mechanic	Isocyanates
	Dentist	Dental technician	Acrylate compounds
**Metal**	Hard metal lung	Polishing	Cobalt, tungsten

Abbreviation: HP, hypersensitivity pneumonitis.

Sources: *Respiratory Investigation* 2024; 62: 16-43; *Respiratory Medicine* (Japanese) 2020; 38: 124-130.

**Table 3:** Classification of hypersensitivity pneumonitis based on source.

**Source**	**Specific environment**	**Name of disease**
Water pollution	Becomes a microbial bioaerosol emitter	
Ventilation	Contaminated water in humidifiers and air conditioners, air conditioners with water tanks (cold air blowers and fans), dehumidifiers	Humidifier lung
Living environment	Flooding of houses; slippery and moldy parts in bathrooms, kitchens, and other wet areas; contamination of wooden furniture, tatami mats, carpets, sofas, and other upholstered furniture; water pipes; moisture and mold in and around hot tubs (recirculating and jetted tubs); and contamination of saunas	Summer-type HP, home-related HP, wind-instrument alveolitis, bagpipe lung, hot tub lung, and sauna taker’s lung
Occupational	Contamination of metal-working fluids (cooling and lubricating water), sewage treatment, and plumbing in automotive and other plants	Machine operator’s lung, sewer worker’s lung
**Plant-related**		
Agriculture and food processing	Moldy hay, grain, and stored grass; sugar cane, sugar mills, and confectionery manufacturing; tobacco; mushroom spores and compost for mushroom cultivation; orchids, roses, cucumbers, and other greenhouse crops; compost; mold on potatoes, bell peppers, grapes, and other vegetables and fruits; breweries; coffee bean dust; tea growing; and green tea powder	Farmer’s lung, bagassosis, tobacco grower’s lung, mushroom grower’s lung, compost lung, potato sorter’s lung, paprika slicer’s lung, wine grower’s lung, coffee worker’s lung, and tea grower’s lung
Grain	Grain flour (wheat, malt, beans, etc.), flour, dried bonito flakes, cheese, and others contaminated with corn mites (flour mites); weevil-infested flour; moldy barley; bakery and bread making yeast; and pastry	Flour-dust alveolitis, miller’s lung, wheat-weevil lung, malt worker’s lung, and baker’s lung
Wood	Timber harvesting, maple bark stripping, wood chips and dust from woodworking including hobbies (DIY), wood shavings, sequoia sawdust, wood pulp, dry rot in wood, fallen leaves, dead leaves, thatched roofs, and cork mold	Wood trimmer’s disease, maple bark disease, woodworker’s lung, wood fiber alveolitis, sequoiosis, wood pulp worker’s disease, dry rot lung, thatched-roof lung, and cork worker’s lung
Other	Stains on painted exterior plaster walls, cosmetic powders (argan cake), and food waste	Stucco worker’s lung, argan cake HP, and waste sorter’s lung
**Animal-related**		
Birds	Birdkeeping, use of feather products (duvets, pillows, and feather coats), contact with bird excrement and feathers, and use of poultry manure fertilizers	Bird fancier’s lung, feather-duvet lung, and bird-related HP
Animals other than birds	Animal fur, hair and excrement from laboratory animals (rats and other rodents), bat excrement, cocoon scraping and debris removal in sericulture, and foot care	Furrier’s lung, laboratory worker’s HP, sausage worker’s lung, bat lung, silkworm rearer’s lung, and foot care alveolitis
Food processing	Cheese mold, sausage production, fish meat and fish meal dust, oyster shell powder, and milk (infant)	Cheese washer’s lung, salami producer’s lung, fish food lung, fish meal worker’s lung, oyster-shell HP, and Heiner syndrome
**Industrial (inorganic)**		
Chemicals	Isocyanates (spray paint for polyurethane foam, rubber, lacquer paint, automotive maintenance, castings), acid anhydride curing agents such as epoxy resins in plastic processing, acrylic resins for dental materials, Bordeaux solution (pesticide containing copper sulfate and other chemicals used on grapes), coolants for laser hair removal, and polyester powder (paint)	Isocyanate alveolitis, acid anhydride alveolitis, methacrylate alveolitis, vineyard sprayer’s lung, hair-remover lung, painter’s lung
Metals	Zinc fumes, cobalt, zirconium, trimethylindium, beryllium	Giant cell pneumonitis, zinc-fumes alveolitis, and beryllium HP
Drugs	Penicillins, cephems, methotrexate, interferon α, pravastatin, etc.	Drug-induced HP

Abbreviation: HP, hypersensitivity pneumonitis.

Source: Institute of Science Tokyo (https://www.tmd.ac.jp/med/pulm/hp.html) [in Japanese].

### 2.2. Genetic factors

This disease is mainly caused by an allergic reaction. Therefore, genetic polymorphisms in the human leukocyte antigen (HLA) haplotype and the promoter region of cytokine genes are investigated as disease susceptibility genes. HLA-DR27 has been frequently detected in bird-related HP of Mexicans,^11^ HLA-B8 has been frequently detected in bird-related HP and farmer’s lung of Caucasians,^12^ and HLA-DQw3 has been frequently observed in summer-type HP of Japanese.^13^ Regarding cytokine genes, a significant number of patients have been found to have the A2 allele in the promoter region 308 of tumor necrosis factor-alpha.^14^ These are reports about acute HP. Conversely, 17.5% of patients with chronic subclinical onset HP had a family history of interstitial pneumonia. Since they did not live in the same environment, genetic factors may play a role in the onset of chronic disease in Japan.^6^ Recently, 1 single nucleotide polymorphism (SNP) in the mucin 5B gene, rs35705950, and 3 neighboring SNPs in the toll-interacting protein (TOLLIP) variants (rs3750920, rs111521887, and rs5743894) have been reported to be associated with increased susceptibility to fibrosing HP (chronic HP) in Portugal.^15^ The TOLLIP gene, rs5743899 GG genotype, has been identified as a potential facilitating factor for rapid lung capacity decline in chronic HP in Japan.^16^ These findings indicate that HP is affected by the genetic background of patients.

## 3. Diagnosis

Identification and avoidance of causative antigens are considered important in the prognosis of HP.^17^^,^^18^ However, these are often difficult. For the lack of reliable antigen identification tests, the procedures of antigen detection include estimation by medical interviews, environmental provocation tests, and so on.^2^^-^^4^ In Japan, there are only 2 types of commercially available tests, for *Trichosporon asahii* and for bird-related antigens (pigeon and budgerigar).^3^ Each attempts have been made to detect various causative antibodies using the Ouchterlony method, enzyme-linked immunoassay, and other methods, they are done in each laboratory. Due to a lack of standardization of measurement methods, each test results cannot be compared. The 2020 ATS/JRS/ALAT guidelines for HP highlight the need to establish internationally standardized panel tests to detect causative antigens.^2^ The usefulness of lymphocyte stimulation tests with antigens has also been reported in part, but it has not been established.^19^ Although not specific, seasonal and environmental variations in biomarkers such as KL-6 (sialylated carbohydrate antigen KL-6) and Sp-D (surfactant protein D) and the increase of lymphocytes in bronchial alveolar lavage fluid may be helpful.^20^^,^^21^ In other words, HP is diagnosed by comprehensively evaluating the clinical phenotype, environment of onset, imaging/pathology, and immunological findings. For the final diagnosis, a multidisciplinary discussion (clinician, radiologist, and pathologist) should be considered. On the clinical side, assessing antigen exposure through interviews and environmental surveys in the workplace is important. Additionally, gathering information from occupational physicians and hygiene specialists in the workplace is necessary. The antigen exposure evaluation form from the Institute of Science Tokyo is a useful tool for collecting patient information (Table 4).

**Table 4:** Antigen questionnaire (Institute of Science Tokyo).

**Below is a questionnaire on the possible environmental causes of lung disease. Please fill out the following questionnaire or tick the appropriate box.**
(1) Present address (*city/ward/town/village in metropolitan/provincial/prefectural government*)
(2) Year of construction () (*reinforced concrete, wood-frame*) (*detached house, apartment building, other*) ()
(3) Room with poor lighting: (Yes or No; if yes, which room specifically?)
(4) Room with high humidity: (Yes or No; if yes, which room specifically?)
(5) Damp room: (Yes or No; if yes, which room specifically?)
(6) Smell of mold: (*Yes or No*)
(7) Mold: (*Not noticeable, Yes*) Areas with mold growth (such as *bathroom, washing machine, washroom, kitchen, window, wall, wooden furniture, tatami, futon, or carpet:*)
(8) Do you have a *whirlpool, bathtub or jacuzzi, are you accustomed to storing bath water, or do you use a mist sauna*? (*Yes or No*)
(9) Have you ever had any leaks or flooding in your house? (*Yes or No*)
(10) Do you have any *fields, rivers, or wetlands near your house*? (*Yes or No*)
**Questions about your living environment:**
(1) Do you use a humidifier? (Use: in what season?; Do not use)
(2) Do you use an air conditioner, and how long has the oldest one been in use?
(Use: indicate approx. years since purchase; Never used)
(3) Do you use a feather quilt: (Yes; Used to; Never used)
Do your family members who live with you also use feather quilts: (Yes; Used to; Never used)
(4) Do you have any other bird-related products in your home:
(No; feather coat; feather pillows; feather dippers; bird skins; other:_____________)
(5) Do you use poultry manure for gardening at home or in your neighborhood:
(I don’t; I use it at home; My neighbors use it; I don’t know)
(6) Did you raise birds, including as a child (Did you keep birds or not?)
When, what species, and how many birds did you keep?
(__________________________________________)
(7) Are there any houses keeping birds, aviaries, birdhouses, or bird nests in the neighborhood: (Yes; No; Don’t know)
(8) Do you see birds flying, feathers, or droppings in your yard or on your balcony (Yes; No)
(9) Do you feed birds in your garden or park: (Yes; No)
(10) Do you currently keep animals other than birds as pets: (Yes; No)
**Please describe your work history.**
From *year to year (Name of occupation)* (Specifically, Ex: [occupation])
From *year to year (Name of occupation)* (Specifically,)
From *year to year (Name of occupation)* (Specifically, )
**We would like to ask you about your *business/hobby that involves the inhalation of mold or dust*. Please check if you engage in *any of the following on a daily basis*.**
□ Farming, growing vegetables, fruits, flowers, mushrooms
□ Hay, livestock feed, compost
□ Priming/planting garden trees
□ Vegetable sorting
□ Grain for bread, confectionery, and noodle making, among others
□ Tea and coffee bean powder
□ Food processing (sausage and cheese making, among others)
□ Cleaning up fallen leaves and garbage
□ Animal hair and excrement
□ Wood chips, wood dust, and DIY
□ Moldy cork, lumber, and bark
□ Wind instruments (such as saxophone)
□ Cooling water and lubricating oil in machinery
□ Metal dust (welding and grinding)
□ Paints and sprays containing isocyanate
□ Cold air conditioners with water tanks, cold fans, and dehumidifiers
(polyurethane foam and lacquer, among others)
Please describe any other jobs or hobbies that may cause you to inhale mold or dust.
(__________________________________________________________)
Of the above, what do you think triggers your symptoms (cough, breathlessness, slight fever)?
(__________________________________________________________)

Source: https://www.tmd.ac.jp/med/pulm/hp.html (in Japanese).

### 3.1. Clinical features

Acute HP manifests within several weeks to months, with symptoms such as coughing, shortness of breath on exertion, fever, and general fatigue. Symptoms are often clearly related to antigen exposure and appear as influenza-like symptoms 4-12 hours after exposure. Chronic HP rarely presents with acute symptoms. Patients present with dyspnea on exertion, general fatigue, anorexia, cough, and weight loss, which gradually progress over several months to several years (Table 5). Summer-type HP develops and worsens in summer, whereas humidifier lung and bird-related HP caused by feather products develop and worsen in winter. In chronic HP, fine crackles are heard on chest auscultation in 90% of the cases, and clubbing is observed in 30% of the cases.^6^ Fine crackles are a physical finding common to idiopathic pulmonary fibrosis (IPF) and other fibrotic lung diseases, but squawk during inspiration suggests the presence of bronchiolar disturbance and is a characteristic finding of this disease. Patients exhibit hypoxemia, especially during exertion. A 6-minute walk is effective for evaluating disease progression. Regarding laboratory findings, KL-6 and SP-D are markedly elevated in acute HP but moderately elevated in chronic HP. Pulmonary function tests show restrictive disturbance.

**Table 5:** Clinical features of hypersensitivity pneumonitis.

	**Acute HP**	**Chronic HP**
**Antigen exposure**	Short time exposure to high concentration of antigens (summer-type HP, farmer’s lung)	Continuous exposure to low concentration of antigens (bird-related HP)
**Onset form**	Course of several weeks to months, cough, shortness of breath during exertion, fever, general malaise, etc.	Course of months to years, difficult to distinguish from IPF
**Symptoms**	The association with antigen exposure is often obvious, with symptoms occurring 4-12 h after exposure	Gradual progression of dyspnea on effort, general fatigue, anorexia, cough, and weight loss
**Physical findings**	Fever, fine crackles	Fine crackles (90%), club finger(30%), inspirational squawk
**Prognosis**	Good, steroids effective	UIP pattern: 5-year survival rate is 50%-70%

Abbreviations: HP, hypersensitivity pneumonitis; IPF, idiopathic pulmonary fibrosis; UIP, usual interstitial pneumonia.

### 3.2. Environment of onset

When detecting the causative microorganisms in the environment, *Trichosporon*, which causes summer-type HP, is frequently detected in Japan. This mold tends to breed in damp environments, such as rotten wood, damp or aquatic areas, and rain leaks in homes and workplaces. A swab and culture of liquid from the suspected parts is needed to detect this mold. Additionally, microbial air sampling is effective. It is beneficial for inspectors to check for the arrival of birds, breeding of birds, down comforters, down jackets, use of chicken fertilizer, and presence of bird taxidermy. Furthermore, checking not only the workplace but also the surrounding environment is necessary. Table 3 shows the other main circumstances under which OHP develops.

### 3.3. Imaging/pathology

**Figure 1:**
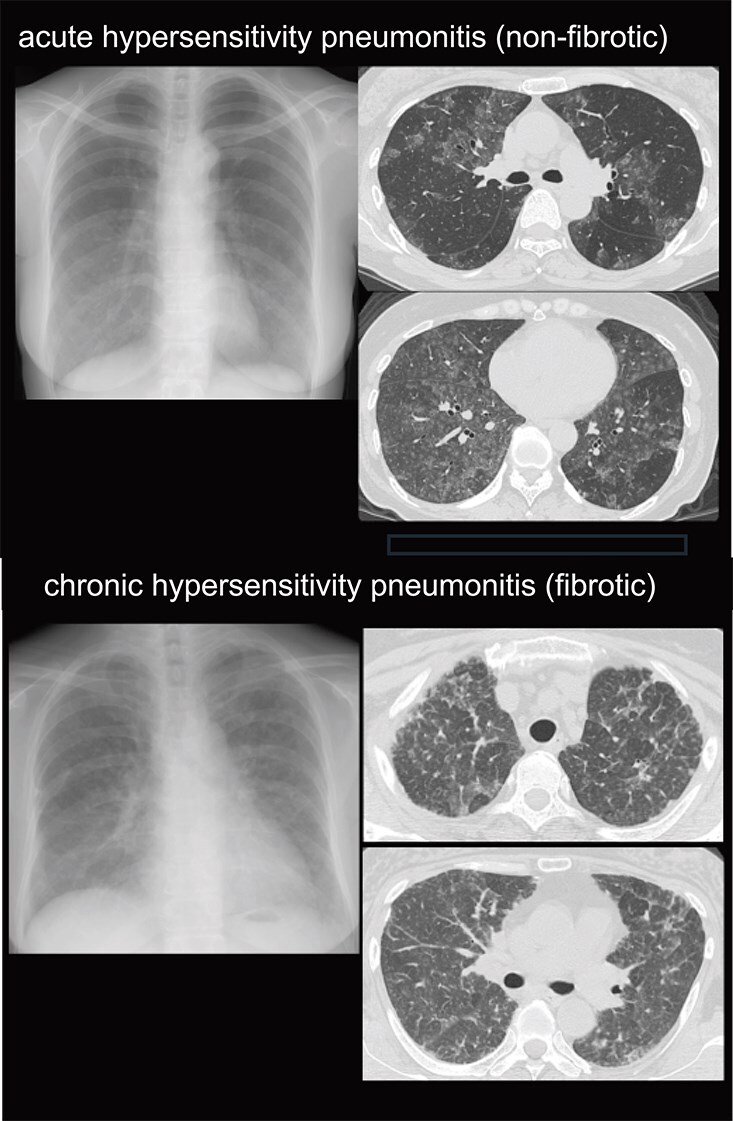
Imaging of acute hypersensitivity pneumonitis (upper) and chronic hypersensitivity pneumonitis (lower). Images were provided by the Institute of Science, Tokyo Hospital.

Diagnostic imaging is generally performed by HRCT (high-resolution computed tomography). In the 2020 ATS/JRS/ALAT guidelines, in accordance with the international guidelines for IPF, images are taken in the supine position and during deep inspiration, captured as continuous slice data with a thickness of 0.5-1 mm. The data are reconstructed to visualize a 5-mm thick CT (lung field and mediastinal condition) and 0.5-1.25-mm thick HRCT.^2^^,^^22^ On HRCT, acute (nonfibrotic) HP presents with centrilobular granular shadows, small nodules with unclear margins, and panlobular ground-glass opacities, and it sometimes shows a mosaic distribution. Ground-glass shadows have varying degrees of dark and light areas and are occasionally near the consolidation shadow (Figure 1). Chronic (fibrotic) HP presents with diverse imaging findings (Figure 1). The distribution is predominantly in the upper lung field or in both the upper and lower lung fields. Advanced cases present with honeycombing and are difficult to differentiate from IPF.

The pathological findings of acute HP exhibit 3 characteristics: (1) cellular bronchiolitis, (2) nonnecrotizing granuloma in the lung interstitium, and (3) lymphocytic inflammation in the lung interstitium with almost no alteration of the existing lung structure. Chronic cases exhibit various histopathological patterns, including organizing pneumonia, nonspecific interstitial pneumonia (NSIP), and usual interstitial pneumonia (UIP). Centrilobular and bridging fibrosis are other important pathological findings indicative of exposure to inhaled antigens.^23^

### 3.4. Immunological findings/environmental provocation test/antigen provocation test

The detection of specific antibodies is diagnostically useful. The anti-*Trichosporon asahii* antibody test is useful for summer-type HP, and the bird-related antibody test is useful for bird-related HP, both of which are approved by the National Health Insurance in Japan.^24^^,^^25^ The sensitivity and specificity of the antibodies are >90% for acute HP and approximately 50% and 80%, respectively, for chronic HP.^3^ Environmental provocation tests in the workplace are commonly used to diagnose OHP. Although no criteria for evaluation items have been established to compare the situation of the patients before and after exposure to antigens, systemic symptoms such as chills and general fatigue, respiratory symptoms such as cough and shortness of breath, fever, high white blood cell count, elevated C-reactive protein level, shadows on chest x-ray and CT, increased A-aDO_2_ (alveolar-arterial oxygen difference) on arterial blood gas analysis, and decreased vital capacity and diffusing capacity for carbon monoxide on pulmonary function tests may provide helpful information for diagnosis. In some cases, clinical symptoms became apparent within 1 week to 1 month despite not showing any positive results for diagnosis immediately after exposure. In the case of diisocyanates, the antigen inhalation provocation test more precisely proves that they are causative antigens. However, it is unlikely to be conducted in Japan. Another testing method has been reported in which subjects are exposed to diisocyanates (concentration 5-20 ppb) indoors for a certain period of time.

## 4. Differential diagnosis

The differential diagnosis includes the following: (1) drug-induced pneumonia (differentiation of acute HP): the condition improves when the drug is discontinued and relapses when the drug is readministered; (2) IPF (differentiation of chronic HP): it is difficult to differentiate from the UIP pattern of chronic HP; (3) NSIP (differentiation of chronic HP): it is difficult to differentiate from the NSIP pattern of chronic HP; and (4) common cold, acute respiratory infection, and atypical pneumonia (differentiation of acute HP): it is important to suspect the possibility of HP as a differential diagnosis. The diagnosis can be made through detailed interview, physical examination, imaging, blood tests, and the ineffectiveness of antibiotics, etc.

## 5. Complications

Acute HP can be complicated by acute respiratory failure. Chronic HP can be complicated by chronic respiratory failure, pulmonary hypertension, right heart failure, pneumothorax, pneumomediastinum, and respiratory infections, including opportunistic infections. Additionally, acute exacerbations occur annually in 10% of patients with chronic HP.^26^^,^^27^

## 6. Disease course and prognosis

Acute HP is often relieved by antigen avoidance. Severe cases complicated by respiratory failure are often relieved by steroid treatment. Chronic HP progresses relatively slowly and often follows a course similar to that of IPF.^28^ Progressive pulmonary fibrosis determines the prognosis of this disease. Accurate antigen avoidance can slow down the progression of fibrosis. However, antigen avoidance is often difficult, and even with drug treatment, cases showing a UIP pattern are progressive and have a poor prognosis. The 5-year survival rate of patients with chronic HP accompanied by UIP is 50%–70%, which is similar to IPF.^29^^-^^31^

## 7. Treatment and prevention

Antigen avoidance is the fundamental treatment of HP. In painter’s lung, exacerbation may persist for approximately 1 week even after leaving the workplace, necessitating caution. Controlling allergic inflammation and suppressing fibrosis at an early stage using steroids, immunosuppressants and antifibrotic drugs are important. However, these effects are sometimes limited especially in chronic cases.^32^^-^^34^ Once fibrosis sets in, treatment becomes difficult.

### 7.1. Acute HP

Antigen avoidance and environmental improvements are crucial to treating and preventing acute HP. For summer-type HP, environmental improvements, including building renovations, are necessary. The company manager should pay particular attention to *Trichosporon* and dispose of rotting wood, bedding, tatami mats, and carpets. If conditions do not improve, workers with the disease should be rearranged or relocated. In bird-related HP, bird keeping should be discontinued, and feather duvets and down jackets, including those of colleagues and family, should be discarded. If avoiding bird-rich environments, such as stations, parks, and shrines, is impossible, individuals should consider rearrangement, relocation, and moving. Workers should wear appropriate dust masks to prevent the occurrence of farmer’s and painter’s lung. For humidifier lung, individuals should regularly replace the filters and thoroughly clean the equipment. Mild cases may improve with antigen avoidance alone. Moderate to severe cases accompanied by respiratory failure can be treated with steroid therapy.

### 7.2. Chronic HP

Antigen avoidance is mandatory. Chronic HP (fibrotic HP) resistant to antigen avoidance has a poor prognosis. In cases of fibrosis progression or severe respiratory failure, long-term steroid therapy is required with or without immunosuppressive therapy. The effectiveness of antifibrotic drugs has been reported in progressive fibrotic cases.^35^ However, there are many cases in which antifibrotic drugs are not effective, and as with other interstitial lung diseases, there are large unmet needs for new therapeutic agents. Early antigen identification and antigen avoidance are important to improve the prognosis of chronic HP.^36^^-^^39^

## 8. Conclusion

The significance of OHP has gradually increased. Generally, HP has a poor prognosis when it becomes chronic. Antigen avoidance is the cornerstone of treatment for OHP. The antigen remains unidentified in many cases of OHP, and industrial physicians should consider the possibility of antigen exposure, such as the working process and timing of onset, when seeing interstitial lung disease in workplaces. Currently, exposome research on HP to clarify the overall picture of exposure from the internal and external environment of an individual is being attempted. Further studies using various approaches are needed to provide more pathological information about OHP. An internationally standardized panel test for specific antigens and more effective treatments for fibrosis cases are needed as soon as possible. In reality, it will take several years to realize these things. In order to improve the prognosis of this disease now, it is important for occupational physicians and hygiene specialists to be aware of the existence of this disease and to halt its progression through early medical interviews, environmental surveys, and referral to a pulmonologist.
