# Correction to: Importance of early detection and treatment of occupational hypersensitivity pneumonitis

**DOI:** 10.1093/joccuh/uiaf033

**Published:** 2025-06-21

This is a correction to: Shinya Ohkouchi, Yasuo Morimoto, Narufumi Suganuma, Hajime Kurosawa, Kenichi Azuma, Hisamitsu Omori, Taro Tamura, Kunio Dobashi, Kengo Nakamoto, Makiko Nakano, Yuji Natori, Naomi Hisanaga, Kiyoshi Mizushima, Kazuhiro Yatera, Yasunari Miyazaki, on behalf of Japan Society for Occupational Health, Occupational Lung Disease Study Group, Importance of early detection and treatment of occupational hypersensitivity pneumonitis, *Journal of Occupational Health*, Volume 67, Issue 1, January-December 2025, uiaf026, https://doi.org/10.1093/joccuh/uiaf026

The following changes have been made to the originally published manuscript. In the author list, the name of group: “on behalf of the Occupational Respiratory Disease Study Group in Japan” has been emended to: “on behalf of Japan Society for Occupational Health, Occupational Lung Disease Study Group”.

